# MAIT cells accumulate in placental intervillous space and display a highly cytotoxic phenotype upon bacterial stimulation

**DOI:** 10.1038/s41598-017-06430-6

**Published:** 2017-07-21

**Authors:** Martin Solders, Laia Gorchs, Tom Erkers, Anna-Carin Lundell, Silvia Nava, Sebastian Gidlöf, Eleonor Tiblad, Isabelle Magalhaes, Helen Kaipe

**Affiliations:** 1 Division of Therapeutic Immunology, Department of Laboratory Medicine, Karolinska Institutet, Karolinska University Hospital, SE-141 86 Stockholm, Sweden; 2 Department of Rheumatology and Inflammation Research, Sahlgrenska Academy University of Gothenburg, Gothenburg, Sweden; 3 Department of Obstetrics and Gynaecology, Karolinska University Hospital, Huddinge, SE-141 86 Stockholm, Sweden; 4 Department of Clinical Science, Intervention and Technology, Karolinska Institutet, Karolinska University Hospital, Huddinge, SE-141 86 Stockholm, Sweden; 5 Department of Women’s and Children’s Health, Karolinska Institutet, SE-171 77 Stockholm, Sweden; 6 Department of Oncology-Pathology, Karolinska Institutet, SE-171 77 Stockholm, Sweden; 7 Department of Clinical Immunology and Transfusion Medicine, Karolinska University Hospital, Huddinge, SE-141 86 Stockholm, Sweden

## Abstract

During pregnancy, the maternal immune system must tolerate the developing foetus, and yet retain a potent antimicrobial response to prevent infections. Mucosal associated invariant T (MAIT) cells recognize microbial-derived vitamin B metabolites presented on the MR1 molecule, but their presence and function at the foetal-maternal interface is not known. We here isolated mononuclear cells from paired samples of peripheral blood (PB), intervillous blood (IVB), and decidua parietalis (DP) following uncomplicated term pregnancies. Interestingly, MAIT cells were highly enriched in IVB compared to PB and DP. The activation status of IVB MAIT cells was similar to that of PB MAIT cells, except for a lower expression of PD-1. Both IVB MAIT cells and conventional T cells were more dominated by an effector memory phenotype compared to PB MAIT cells and T cells. IVB MAIT cells also responded more vigorously with expression of IFN-γ, granzyme B, and perforin in response to *Escherichia coli* stimulation compared to PB. MR1 was not expressed in syncytiotrophoblasts, but in placental villous and decidual macrophages. These data indicate that maternal MAIT cells accumulate in the intervillous space of the placenta and that they are highly armed to quickly respond if bacteria are encountered at the foetal-maternal interface.

## Introduction

During pregnancy, the maternal immune system is capable of recognizing the foetal semi-allogeneic antigens^1^. However, a detrimental immune response is still absent even though maternal peripheral lymphocytes react vividly against foetal antigens *in vitro* ^2^. This is due to several tolerance mechanisms at the foetal-maternal interface^3^, as well as the presence of specialized foetal trophoblast cells which lack the expression polymorphic HLA molecules^4^. This provides a tolerogenic milieu that protects the foetus from rejection^5^.

Pregnant women can be more severely affected by some infections, including influenza, *Listeria* and malaria, than non-pregnant women^6^. This likely reflects the alteration of the immune system of the mother during pregnancy, with a decreased T cell mediated immunity and increased proportions of regulatory T cells^6^. For a successful pregnancy, it is crucial that the immune system at the foetal-maternal interface exhibits immunity to microbes while maintaining foetal tolerance^3^.

The decidua is a maternal membrane that differentiates from endometrial cells under the influence of progesterone during the first trimester. The decidua is invaded by foetal extravillous trophoblasts during implantation, which can interact with maternal immune cells infiltrating the membrane. During the first trimester, the majority of decidual immune cells are CD56^high^CD16^−^ NK cells, whereas T cells only constitute about 10% of the CD45^+^ population^7^. However, this change as pregnancy proceeds, with an increased proportion of T cells at term. Another site for maternal immune cell-foetal interaction is the intervillous space, where maternal blood is in direct contact with the syncytiotrophoblasts lining the chorionic villi. The general notion is that the maternal blood volume is replaced 2–3 times every minute to provide exchange of gases and nutrients^8^, but very little is known about the composition and phenotype of immune cells in intervillous blood during healthy pregnancy.

Bacteria and other microorganisms can cross the placental barrier and trigger an inflammatory response, which can cause premature contractions or even rupture of the placental membranes^9^. *In vitro* studies have shown that trophoblasts produce a wide variety of anti-microbial substances^5, 10^ and decidual NK cells are able to control cytomegalovirus (CMV) infections^11^. It has also been shown that memory CD8^+^ T cells specific for CMV and Epstein-Barr virus accumulate in decidual tissues^12^.

Mucosal associated invariant T (MAIT) cells respond to microbial derived vitamin B metabolites^13^, bound to the non-classical MHC class I related molecule (MR1)^14^. MR1 is highly conserved among species, indicating its vital role in host defense^15^. Only microorganisms with a functional riboflavin metabolism can activate MAIT cells^16, 17^, including *Escherichia coli* (*E*. *coli*), *Mycobacteria* and *Lactobacillus* species^16^. MAIT cells are characterized by the expression of the T cell receptor subunit Vα7.2 and the C-type lectin CD161, and are predominantly CD8^+^ T cells, although a small proportion is CD4/CD8 double negative or CD4^+^^18^.

Apart from the MR1-dependent activation, MAIT cells can be functionally activated by stimulation with IL-7, IL-12, IL-15, or IL-18^19, 20^. Upon stimulation, MAIT cells react by secreting interferon-γ (IFN-γ), tumour necrosis factor-α, and IL-17^16, 21^, as well as mediate cytotoxic effects via granzyme B (GrzB) and perforin^22^. Low numbers of systemic MAIT cells have been associated with severe systemic diseases, especially during bacterial infections^17, 23^, and their function has been shown to be impaired in patients with chronic viral infections, such as hepatitis and HIV^24, 25^.

Despite their importance in anti-bacterial defence, the function and presence of MAIT cells in placentas have not been studied previously. This study aimed to characterize the phenotype as well as assay the functionality of MAIT cells at the foetal-maternal interface. We isolated lymphocytes from the maternal blood infiltrating the intervillous space, herein referred to as intervillous blood (IVB), as well as decidua parietalis (DP), maternal peripheral blood (PB), and umbilical cord blood (CB). Interestingly, we found that the composition of immune cells in IVB was different compared to PB, and that MAIT cells were enriched in IVB.

## Results

### The proportion of MAIT cells is increased in intervillous blood

**Figure 1:**
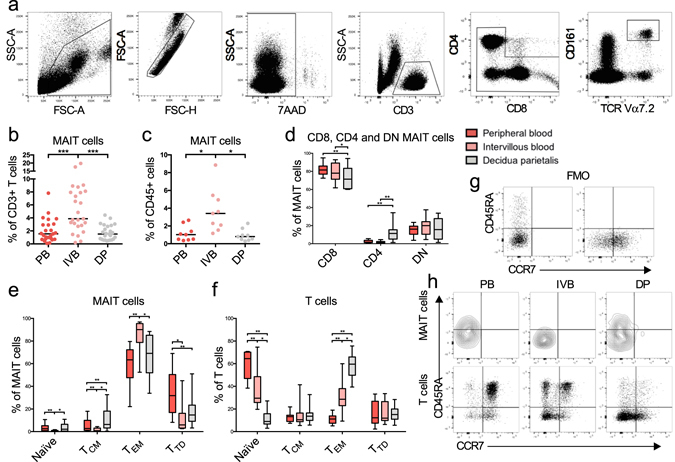
Increased frequency of MAIT cells in intervillous blood. (**a**) Representative flow cytometry plots showing gating strategies to identify CD161^high^ and Vα7.2^+^ MAIT cells. (**b**–**f**) Comparison between paired samples of peripheral blood (PB), intervillous blood (IVB), and decidua parietalis (DP) cells. MAIT cells expressed as percentage of (**b**) CD3^+^, (*n* = 25) and (**c**) CD45^+^ (*n* = 9) cells. (**d**) CD4^+^, CD8^+^, and double negative (DN) MAIT cells expressed as percentage of total MAIT cells (*n* = 11). (**e**) CD45RA^+^CCR7^+^ (Naïve), CD45RA^−^CCR7^+^ (Central memory, T_CM_), CD45RA^−^CCR7^−^ (Effector memory, T_EM_), and CD45RA^+^CCR7^−^ (Terminally differentiated, T_TD_) MAIT cells expressed as percentage of total MAIT cells (*n* = 11) and (**f**) T cells expressed as percentage of total T cells (*n* = 11). (**g**) Fluorescence minus one (FMO) controls for the experiments in (**e**,**f**), and (**h**) representative flow cytometry plots showing gating strategies for Naïve, T_CM_, T_EM_ and T_TD_ MAIT cells (top) and T cells (bottom). Box and whisker plots show median, interquartile range and range of paired samples. The Friedman test followed by the Wilcoxon signed rank test was used to detect statistically significant differences across multiple paired samples (**b**–**f**). A Bonferroni corrected p value was used. *P < 0.05, **P < 0.01, ***P < 0.001.

We first examined if MAIT cells were present at the foetal-maternal interface. MAIT cells were distinguished by excluding CD4/CD8 double positive T cells from single, live CD3^+^ lymphocytes and further identified as CD161^high^ and Vα7.2^+^ (Fig. 1a). The proportion of MAIT cells was consistently higher in IVB, with a median level of 3.9% of the total CD3^+^ T cell population compared to 1.6% and 1.5% in PB and DP, respectively (Fig. 1b). Furthermore, MAIT cells were also enumerated in IVB compared to PB and DP when analysing their proportion within the total CD45^+^ population (Fig. 1c).

As previously shown by others, the majority of the MAIT cells were CD8^+^ and the proportions were similar between the different compartments (Fig. 1d). However, decidual MAIT cells were CD4^+^ to a higher degree compared to MAIT cells from PB and IVB.

In CB, the proportion of MAIT cells among CD3^+^ T cells was very low, with a median of 0.09% (Supplementary Fig. S1a). The majority of both CD3^+^ T cells and MAIT cell were naïve in CB, as previously shown by Gold *et al*.^26^, but in IVB these cells displayed a memory phenotype (Supplemental Fig. S1b and c), Accordingly, results from chimerism analysis showed that the vast majority of cells in IVB were of maternal origin (Supplementary Fig. S1d). To further verify the origin of the MAIT cells, we enriched CD161^+^ T cells by bead selection. Chimerism analysis showed that an enrichment of CD161^+^ T cells (and MAIT cells) led to a decrease in the amount of contaminating fetal DNA (Supplementary Fig. S2a–d), indicating that the IVB MAIT cells were of maternal origin. Due to the low number of MAIT cells in CB, this compartment was not further included in the study.

### Intervillous MAIT cells and conventional T cells display a stronger effector memory phenotype compared to peripheral blood

Most MAIT cells expressed an effector memory phenotype (T_EM_), a trait most distinguished in IVB with a median of 90% with a CD45RA^−^CCR7^−^ phenotype (Fig. 1e, and gating strategy in Fig. 1g,h). The proportion of MAIT cells with a central memory phenotype (T_CM_, CD45RA^−^CCR7^+^), a terminally differentiated phenotype (T_TD_, CD45RA^+^CCR7^−^), and a naïve phenotype (CD45RA^+^CCR7^+^) was lower in the IVB compared to PB (Fig. 1e).

The proportion of conventional CD3^+^ cells with an T_EM_ phenotype was greatly increased in the placental samples compared to PB, particularly in the decidua but also in the intervillous space, with an inverted proportion of cells expressing a naïve phenotype (Fig. 1f). Thus, both MAIT cells and conventional T cells in the intervillous space had acquired an T_EM_ phenotype to a higher extent than their systemic peripheral counterparts.

Other differences in cell composition between PB and IVB were observed, with a higher proportion of CD19^+^CD20^+^ B cells, CD56^dim^ and CD56^high^ NK cells, as well as CD3^+^CD56^+^ NKT-like cells in IVB compared to PB (Table 1). As expected, DP contained the highest proportion of CD56^high^ NK cells, which previously has been described to be increased in first trimester decidual tissues^27^, but it also contained more CD3^+^CD56^+^ NKT-like cells than PB (Table 1).

**Table 1:** Composition of immune cell subsets in peripheral blood, intervillous blood, and decidua parietalis.

Subpopulation	n	Peripheral blood (PB)	Intervillous blood (IVB)	Decidua parietalis (DP)	Friedman test, *p*	Wilcoxon test, *p* × 3		
						PB vs IVB	PB vs DP	IVB vs DP
T cells (CD45^+^/CD3^+^)	10	63.7 (54.3–71.5)	58.5 (30.2–70–4)	61.7 (51.1–77.7)	0.601			
CD4^+^ T cells (CD3^+^/CD4^+^)	11	65.4 (40.0–82.6)	52.8 (26.0–73.5)	46.9 (38.1–59.5)	0.087			
CD8^+^ T cells (CD3^+^/CD8^+^)	11	27.2 (14.3–54.8)	35.3 (19.4–55.9)	45.4 (30.7–57.0)	0.117			
Double negative T cells (CD3^+^/CD4^−^CD8^−^)	11	5.0 (1.4–14.0)	10.9 (3.5–17.5)	5.1 (2.5–23.4)	**0**.**002**	0.162	0.99	0.162
B cells (CD45^+^/CD19^+^CD20^+^)	9	5.1 (3.6–11.6)	12.4 (3.8–28.7)	1.2 (0.4–5.4)	<**0**.**0001**	**0**.**023**	**0**.**012**	**0**.**012**
Monocytes/macrophages (CD45^+^/CD14^+^)	9	23.2 (13.2–26.8)	15.9 (5.8–45.7)	6.3 (4.0–14.5)	**0**.**031**	0.99	**0**.**012**	0.294
Classical NK cells (CD45^+^/CD56^dim^ CD3^−^)	10	3.7 (1.4–6.1)	4.6 (2.2–18.3)	2.8 (2.0–3.7)	**0**.**0047**	**0**.**012**	0.902	**0**.**012**
Non-classical NK cells (CD45^+^/CD56^high^ CD3^−^)	10	0.5 (0.3–2–0)	1.1 (0.4–2.3)	18.8 (8.6–30.4)	<**0**.**0001**	**0**.**006**	**0**.**006**	**0**.**006**
NKT-like cells (CD45^+^/CD56^+^ CD3^+^)	10	2.2 (0.8–5.4)	3.9 (0.9–9–7)	5.9 (2.8–8.6)	**0**.**0020**	**0**.**029**	**0**.**006**	0.194

Subpopulation name, extracellular markers used for identification by flow cytometry and number of paired samples (n). Median values with range of observed values in parenthesis.

Altogether, these data indicate that the immune cell composition in intervillous space is different compared to PB, with an enrichment of MAIT cells, T_EM_, B cells and NK cells.

### Decidual, but not intervillous, MAIT cells and T cells express an activated phenotype

**Figure 2:**
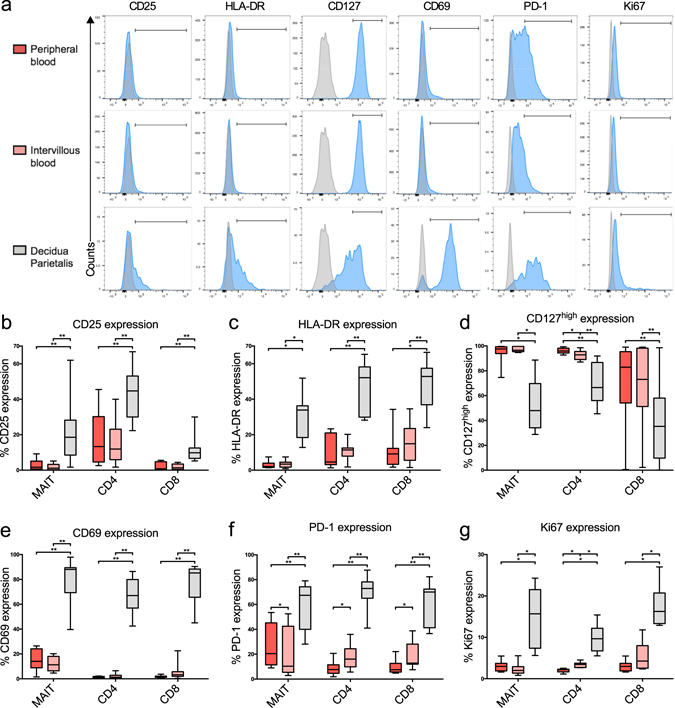
Intervillous blood MAIT cells are not activated, but display a lower PD-1 expression compared to peripheral blood. (**a**) Representative flow cytometry histograms on MAIT cells showing the fluorescent minus one control (grey) and the sample (blue). Bars correspond to the gating strategy which was performed on dot plots. (**b–g**) Comparison between paired samples of peripheral blood (PB) (red), intervillous blood (IVB) (light red), and decidua parietalis (DP) (grey) cells. (**b**) CD25 and (**c**) HLA-DR expression on MAIT cells, CD4^+^ T cells, and CD8^+^ T cells (*n* = 11). (**d**) CD127^high^ expression and (**e**) proportion of CD69 MAIT cells (*n* = 8), CD4^+^ T cells (*n* = 11) and CD8^+^ T cells (*n* = 11). (**f**) Expression of PD-1 in MAIT cells (*n* = 9), CD4^+^ T cells (*n* = 11) and CD8^+^ T cells (*n* = 11). (**g**) Intracellular Ki67 expression in MAIT cells, CD4^+^ T cells, and CD8^+^ T cells (*n* = 8). Box and whisker plots show median, interquartile range and range of paired samples. The Friedman test followed by the Wilcoxon signed rank test was used to detect statistically significant differences across multiple paired samples (**b**–**g**). A Bonferroni corrected p value was used. *P < 0.05, **P < 0.01.

To elucidate whether MAIT cells in placental tissue were locally activated, we evaluated the expression of several activation markers (representative histograms in Fig. 2a). In DP, a large proportion of MAIT cells, as well as conventional CD4^+^ and CD8^+^ T cells (MAIT cells excluded), were positive for the activation marker CD25 (Fig. 2b). A similar pattern was observed for the expression of HLA-DR (Fig. 2c), with decidual MAIT cells and T cells showing an activated phenotype. The proportions of CD127^high^ (IL-7Rα) MAIT cells and conventional T cells were also lower in decidua compared to the other compartments (Fig. 2d). The majority of decidual MAIT cells and conventional T cells expressed the tissue residency marker CD69, whereas the expression of CD69 was low in PB and IVB (Fig. 2e).

The pattern of increased activation in decidual MAIT cells, CD4^+^ and CD8^+^ T cells was accompanied by an increased expression of the inhibitory marker programmed cell death-1 (PD-1) (Fig. 2f). Interestingly, the expression of PD-1 was higher in CD4^+^ and CD8^+^ T cells in IVB as compared to PB T cells, whereas on the other hand the proportion of MAIT cells expressing PD-1 was decreased compared to that of PB. Thus, this indicates that MAIT cells in the intervillous space were less susceptible to inhibitory signals via the PD-1/PD-L1 pathway, with opposing results for conventional T cells.

### Decidual MAIT cells, but not intervillous MAIT cells are proliferating locally

To determine whether the increased proportion of MAIT cells in IVB was due to local proliferation or recruitment, the intracellular expression of the cell division marker Ki67 was measured by flow cytometry. A low Ki67 expression in MAIT cells was observed in both PB and IVB, but the frequency of Ki67 positive cells was higher in DP MAIT cells (Fig. 2g). Decidual CD4^+^ and CD8^+^ T cells also expressed higher levels of Ki67 than PB and IVB. Moreover, a larger proportion of CD4^+^ T cells, but not CD8^+^ T cells, stained positive for Ki67 in IVB compared to PB.

### MAIT cells at the foetal-maternal interface react strongly upon bacterial stimulation

**Figure 3:**
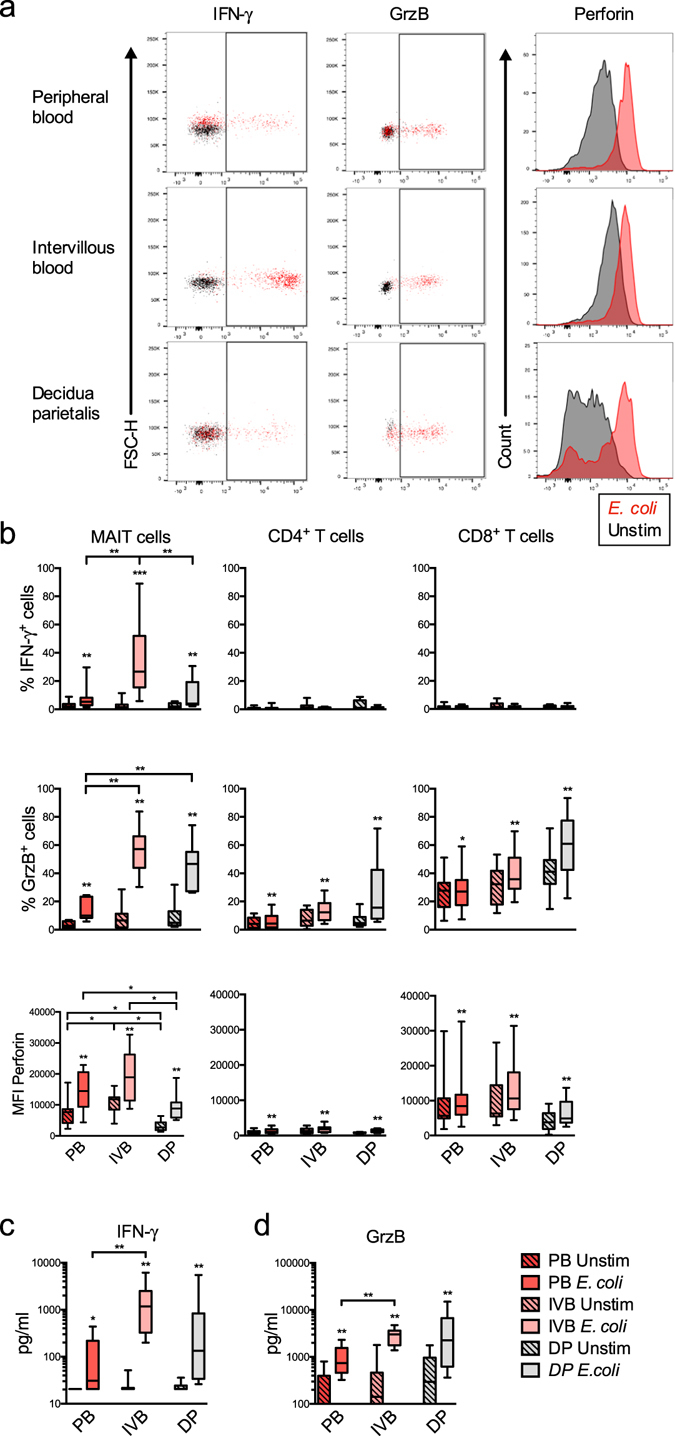
MAIT cells at the foetal-maternal interface react strongly upon bacterial stimulation. (**a**) Representative flow cytometry dot plots and histograms showing the unstimulated sample in black and the *E*. *coli* stimulated sample in red. (**b–d**) Comparison between paired samples of peripheral blood (PB) (red), intervillous blood (IVB) (light red), and decidua parietalis (DP) (grey) cells. The unstimulated condition is shown with diagonal stripes, and the sample stimulated with *E*. *coli* without. (**b**) (Top row) Intracellular expression of IFN-γ in MAIT cells, CD4^+^, and CD8^+^ T cells (*n* = 12). (Middle row) Intracellular expression of Granzyme B (GrzB) in MAIT cells, CD4^+^ and CD8^+^ T cells (*n* = 10). (Bottom row) Intracellular mean fluorescent intensity (MFI) of perforin in MAIT cells (*n* = 9), CD4^+^ (*n* = 10) and CD8^+^ T cells (*n* = 10). Levels of (**c**) IFN-γ, (*n* = 10) and (**d**) GrzB (*n* = 10) measured with ELISA in paired samples of culture supernatants following 16 hours of culture with or without *E*. *coli* stimulation. Box and whisker plots show median, interquartile range and range of paired samples. In **b**, **c** and **d**, the Wilcoxon signed rank test was used to detect statistically significant differences between paired unstimulated and stimulated samples, stars indicate significances. The Friedman test followed by the Wilcoxon signed rank test was used to detect statistically significant differences across multiple paired samples (**b**–**d**). A Bonferroni corrected p value was used for multiple comparisons. *P < 0.05, **P < 0.01, ***P < 0.001.

Previous studies have shown that *E*. *coli-*produced metabolites specifically activate MAIT cells within peripheral blood mononuclear cell cultures^22^. To evaluate whether the response to bacterial stimulation of placental MAIT cells differed from that of MAIT cells from PB and DP, the intracellular expression of the effector cytokine IFN-γ and the cytotoxic proteins GrzB and perforin were measured by flow cytometry after exposing mononuclear cells to PFA-fixed *E*. *coli*. Representative plots and histograms are shown in Fig. 3a.

In unstimulated conditions, the proportion of MAIT cells containing IFN-γ or GrzB was low in all compartments (Fig. 3b). However, the background intensity of perforin expression was higher in IVB and lower in DP compared to PB MAIT cells (Fig. 3b). Interestingly, IVB MAIT cells had a stronger IFN-γ response upon *E*. *coli* stimulation compared to PB and DP MAIT cells (Fig. 3b). Both IVB and DP MAIT cells had a higher expression of GrzB in response to stimulation compared to PB (Fig. 3b). Finally, the MAIT cells from all compartments responded with an increased expression of perforin, and the highest levels were seen in IVB and PB (Fig. 3b). *E*. *coli*-induced IFN-γ production was at large confined to MAIT cells, as the proportion of conventional CD4^+^ and CD8^+^ T cells expressing this cytokine was low (Fig. 3b). The expression of IL-17 and IL-22 in MAIT was undetectable or very low after bacterial stimulation in all compartments in our assays (data not shown).

Secretion of IFN-γ and GrzB by mononuclear cells in response to *E*. *coli* stimulation was also assessed by ELISAs. Supporting the flow cytometry results, the production of both IFN-γ and GrzB was higher in IVB compared to the PB cultures (Fig. 3c and d).

### IL-12p70 and IL-18 levels do not correlate with higher MAIT cell responses in IVB compared to PB

**Figure 4:**
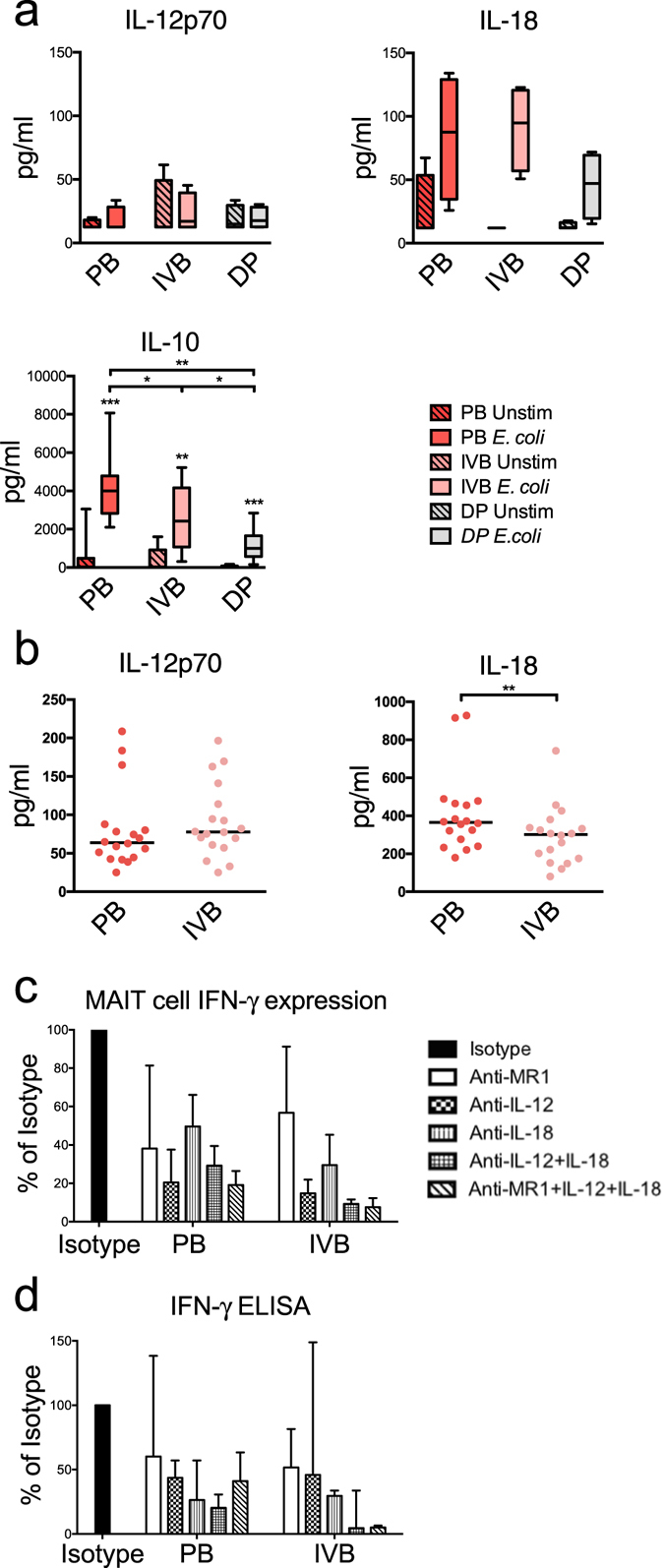
IL-12p70 and IL-18 levels do not correlate with higher MAIT cell responses in IVB compared to PB (**a**) Levels of IL-12p70 (top left, *n* = 4), IL-18 (top right, *n* = 4) and IL-10 (bottom left, *n* = 10) in culture supernatants from paired samples of PB, IVB, and DP following 16 hours of culture with or without *E*. *coli* stimulation. (**b**) Levels of IL-12p70 (left, *n* = 18) and IL-18 (right, *n* = 18) in paired plasma samples from PB and IVB measured with ELISA. (**c**,**d**) The effect of IFN-γ production in paired samples of PB and IVB following 16 hours of culture with *E*. *coli* stimulation after addition of indicated neutralizing antibodies. Data is expressed as percentage cytokine release compared to appropriate isotype control (black). (**c**) Flow cytometric analysis of intracellular MAIT cell expression of IFN-γ (anti-MR1 *n* = 5, anti-IL-12 *n* = 3, anti-IL-18 *n* = 2, anti-IL-12 + IL-18 *n* = 2, anti-MR1 + IL-12 + IL-18 *n* = 3). (**d**) Levels of IFN-γ measured with ELISA on culture supernatants (anti-MR1 *n* = 4, anti-IL-12 *n* = 4, anti-IL-18 *n* = 4, anti-IL-12 + IL-18 *n* = 3, anti-MR1 + IL-12 + IL-18 *n* = 4). Box and whisker plots show median, interquartile range and range of paired samples. In (**a**) and (**b**), the Wilcoxon signed rank test was used to detect statistically significant differences between paired unstimulated and stimulated samples (**a**), or two different groups (**b**), stars indicate significances. The Friedman test followed by the Wilcoxon signed rank test was used to detect statistically significant differences across multiple paired samples (**a**). A Bonferroni corrected p value was used for multiple comparisons. *P < 0.05, **P < 0.01, ***P < 0.001.

Since IL-12 and IL-18 have been shown to be important for activating MAIT cells, we examined the secretion of these cytokines by *E*. *coli*-stimulated mononuclear cells from PB, IVB, and decidua in an attempt to discern the increased MAIT cell response in IVB. The production of IL-12p70 in response to *E*. *coli* was low in all compartments (Fig. 4a), which has been shown previously in PB^28^. IL-18 was produced in all compartments, with similar levels from PB and IVB. On the other hand, IL-10 secretion was higher in PB compared to IVB and decidua. We next examined the levels of IL-12p70 and IL-18 in PB and IVB plasma. There was no significant difference in IL-12p70 levels, but IL-18 was higher in PB compared to IVB (Fig. 4b). Thus, neither the IL-12p70 and IL-18 secretion pattern after *E*. *coli* stimulation, nor the *in vivo* levels of the cytokines appeared to have caused the increased capacity of IVB MAIT cells to produce IFN-γ and GrzB.

Blocking of MR1 reduced the proportion of IFN-γ expressing MAIT cells compared to the isotype control in 5 out of 5 experiments, although at varying efficiency (Fig. 4c). Neutralization of IL-12p40 and IL-18 also had an impact on the IFN-γ response in MAIT cells. The combined addition of anti-IL-12p40, -IL-18, and -MR1 antibodies resulted in a potent blocking of IFN-γ production in 3 out of 3 experiments (Fig. 4c). The intracellular cytokine staining profiles were at large confirmed when analysing IFN-γ levels in cell culture supernatants after blocking MR1, IL-12p40 and IL-18 (Fig. 4d). As expected, little effect was seen on the levels of IL-10 by blocking the various MAIT cell activation pathways (data not shown).

### MR1 is expressed in villous and decidual macrophages, but not in syncytiotrophoblasts

**Figure 5:**
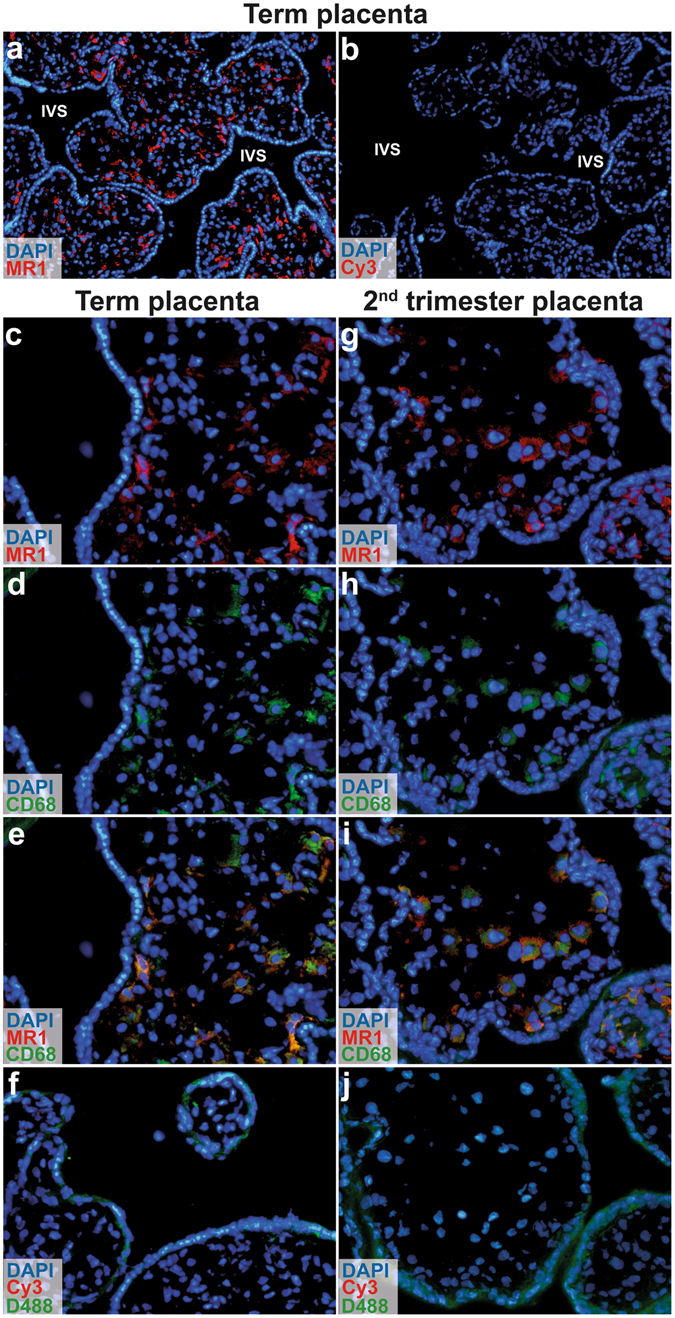
MR1 is expressed by macrophages in foetal villi, but not in syncytiotrophoblasts. Immunofluorescence microscopic images of term placental tissue showing cross sections of chorionic villi and intervillous space (IVS) stained with (**a**) MR1 (red) or (**b**) secondary antibody only (control) at magnification × 10. MR1 was expressed by cells inside the villi, but not in syncytiotrophoblasts. Sections of placental villi at (**c**–**e**) term and (**g**–**i**) gestation week 13 were stained for MR1 (red) and CD68 (green) (×20). (**e** and **i**) Merged images showed that MR1 and CD68 were coexpressed by cells in the villi. Sections stained only with secondary antibody (Cy3) and DyLight488-conjugated (D488) streptavidin complex were used as negative controls (**f** and **j**) (×20). Nuclei were stained with DAPI (blue). The same microscopy settings were kept for the samples and the corresponding controls, *n* = 2 for term placentas and *n* = 1 for 13 week gestation.

We examined the expression pattern of MR1 at the placental barrier using fluorescence microscopy. We observed that the multinucleated layer of syncytiotrophoblasts lining the villi was negative for MR1, whereas subsets of cells inside the villi expressed MR1 (Fig. 5a and b). Double staining of MR1 and CD68 showed that the majority of MR1^+^ cells also expressed CD68, indicating that foetal macrophage-like cells, also termed Hofbauer cells, expressed MR1 (Fig. 5c–f). A similar pattern was observed when analysing the MR1 expression in a placenta from gestation week 13 (Fig. 5g–j), indicating that foetal macrophage-like cells express MR1 also during early pregnancy. Experiments with isotype controls confirmed that both the MR1 and the CD68 stainings were specific (Supplementary Fig. S3).

**Figure 6:**
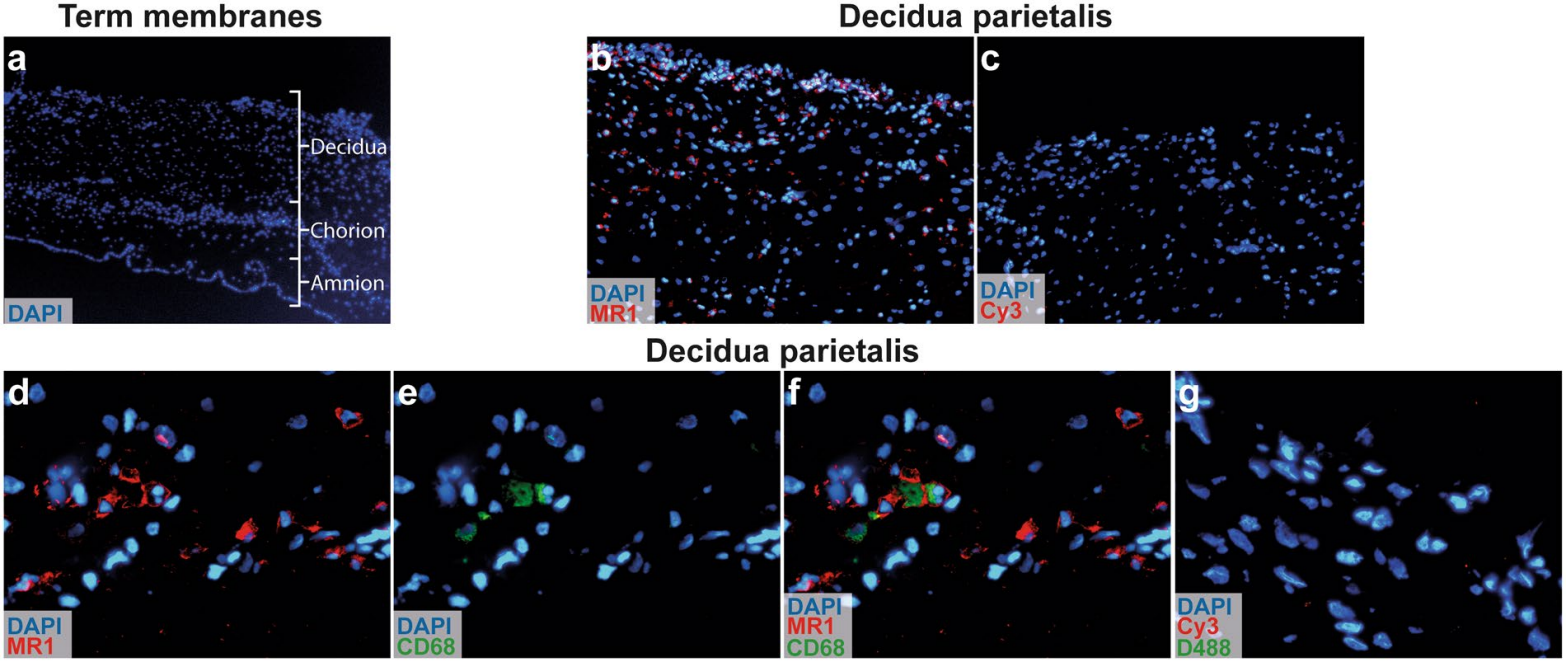
MR1 is expressed by decidual macrophages. (**a**) Immunofluorescence microscopic images of term placental membranes showing cross sections of decidua parietalis, chorion, and amnion at × 4 magnification. (**b**) MR1 staining in the outer decidual layer of the membrane and (**c**) secondary antibody only as control (×10). Decidual tissue stained with (**d**) MR1 antibody (red), (**e**) CD68 antibody (green), and (**f**) merged images showing coexpression of MR1 and CD68 (×40). (**g**) Sections stained only with secondary antibody (Cy3) and DyLight488-conjugated (D488) streptavidin complex were used as negative controls (×40). Nuclei were stained with DAPI (blue). The same microscopy settings were kept for the samples and the corresponding controls, *n* = 2.

We next characterized the MR1 expression in DP. Figure 6a depicts a cross section of the maternal DP and the foetal membranes with DAPI staining. MR1^+^ cells were observed in the decidual layer (Fig. 6b and c). Some, but not all of the MR1^+^ decidual cells coexpressed CD68 (Fig. 6d–g), indicating that decidual macrophages constitutively express MR1.

## Discussion

We here show that MAIT cells are present in IVB and decidua of term placentas delivered after uncomplicated term pregnancies. We further show that MAIT cells are consistently enriched in IVB, that they display an T_EM_ phenotype, and are more responsive to *E*. *coli* compared to MAIT cells from decidual tissues or PB. This indicates that the intervillous space is not only a site for nutrient and oxygen exchange^8^, but also a potential front line of defence against infections.

The composition of decidual immune cells in term placentas has been examined to some extent before^29, 30^, but little is known about the maternal immune cells in the intervillous space. Interestingly, we observed a potent shift from naïve to T_EM_ in IVB compared to PB. This phenomenon was even more pronounced when analysing decidual T cells where naïve T cells were almost absent. On the other hand, the activation status of the T cells in IVB, as determined by CD25 and HLA-DR, was not different compared to PB. This indicates that maternal T_EM_ accumulate in the intervillous space, possibly to provide cell-mediated immunity at the foetal-maternal interface.

Moore *et al*. analysed the general leukocyte populations in IVB compared with PB. They found that the IVB was slightly enriched in monocytes and NK cells, but no other significant differences were seen^31^. In line with these results, we also found that CD56^dim^, as well as CD56^high^, NK cells were enriched in IVB. Vega-Sanchez *et al*. analysed lymphocyte populations from IVB from placentas following both vaginal and caesarean delivery. They observed a lower frequency of T cells in IVB after caesarean section, but not after labour, and a higher frequency of monocytes^32^.

MAIT cells were consistently enriched in IVB, but almost universally found to be in a non-cycling state. The low level of Ki67^+^ MAIT cells suggests that these cells are recruited or retained from the circulating blood rather than locally proliferating. Compartmentalization of MAIT cells in healthy human liver and intestinal mucosa has been previously reported and associated with the presence of commensal bacteria^22, 33^. Recent studies have indicated that placental tissues contain a bacterial community^34, 35^, although this has been questioned by others who failed to distinguish a distinct microbiota when placental tissues from healthy deliveries were compared to a matched set of contamination controls^36^. The low expression of activation markers on IVB MAIT cells suggests that the cells are not in contact with microbial antigens in the absence of clinical symptoms of infection.

The decidua contained similar proportions of MAIT cells as PB, both when analysing the percentage of T cells as well as of total CD45^+^ cells. In contrast, Gibbs *et al*. observed that non-pregnant female genital tract contains lower proportions of MAIT cells compared to PB^37^. It may therefore be speculated that maternal MAIT cells are not only recruited to the intervillous space, but also to the decidua. Given our observation that a proportion of the decidual MAIT cells expressed Ki67, it cannot be excluded that this cell compartment is proliferating in decidual tissues. It is probable that chemokines and endothelial adhesion molecules are involved in MAIT cell recruitment to placental tissues. Further studies of the local chemokines produced from decidual stromal cells and trophoblasts would be of interest to understand the mechanism of MAIT cell accumulation.

The MAIT cells in decidua expressed high levels of the activation markers CD25 and HLA-DR with a concomitant downregulation of CD127 and upregulation of PD-1, indicative of an activated phenotype. The conventional T cells mimicked this expression pattern, a finding supported by other studies of leukocytes in decidua from early and term placentas^7, 29, 30, 38^. These activated T cells in decidual tissues could be involved in defence against pathogens or in controlling trophoblast invasion as well as placental cell growth^38, 39^. Barrier tissues such as skin, gut, lungs and the reproductive tract have been shown to contain a CD69^+^ resident T_EM_ population able to mount a rapid immune response against pathogens^40^. Thus, CD69 is likely a marker for tissue-residency in decidual tissues.

We further found that MAIT cells were not only enumerated in the intervillous space compared to the periphery, but also had a higher expression of intracellular IFN-γ and GrzB when stimulated with a clinical isolate of *E*. *coli*. These results were confirmed when measuring secretion of these factors by ELISA, showing that IVB mononuclear cells produced higher levels of IFN-γ and GrzB compared to PB, although other cell types than MAIT cells could have contributed to the production of these factors. In the unstimulated condition, the expression of perforin was also higher in IVB MAIT cells compared to PB. Decidual MAIT cells also readily expressed IFN-γ, GrzB and perforin upon bacterial stimulation, both intracellularly and when analysing protein secretion.

One possible explanation for the potent expression of cytotoxic molecules by intervillous MAIT cells is their low expression of the co-inhibitory marker PD-1 together with a strong T_EM_ phenotype. In contrast, PB MAIT cells contained a higher proportion of cells with a T_TD_ phenotype, as well as a higher expression of PD-1. Previous studies have shown that the blockade of PD-1 signalling in MAIT cells in patients with active pulmonary tuberculosis resulted in a higher frequency of MAIT cells producing IFN-γ upon bacterial stimulation^41^. An increased PD-1 expression on MAIT cells has also been associated with HIV^42^ and hepatitis C^24^. In contrast to MAIT cells, conventional CD4^+^ and CD8^+^ T cells expressed higher levels of PD-1 in IVB compared to PB. In the decidua, however, both MAIT cells and conventional T cells expressed PD-1 to a higher degree than IVB and PB. The PD-1 ligands PD-L1 and PD-L2 are highly expressed in decidual stromal cells^43^, which possibly could interfere with the immune response *in vivo*.

MAIT cells are activated by TCR interactions with the MR1 molecule, and as shown by many others, blocking of MR1 in our setting decreased the production of IFN-γ in all experiments performed. Since IL-12 and IL-18 also have been shown to be important for activation of MAIT cells, we examined their role in placental MAIT cell activation. The active form of IL-12 (IL-12p70) was low after *E*. *coli* stimulation, which has been shown before in PB mononuclear cells^28^, but blocking of IL-12p40 still had an effect on IFN-γ production from MAIT cells. This could be due to that released IL-12 is rapidly taken up by MAIT cells or other cells expressing the IL-12R. It is also possible that the anti-IL-12p40 antibody interferes with IL-23 signalling, another cytokine that shares the p40 subunit. IL-18 was readily produced from mononuclear cells in all compartments after *E*. *coli* stimulation, and blocking of IL-18 also appeared to have an effect on MAIT cell activation. The plasma levels of IL-18 were higher in PB compared to IVB and no significant difference in IL-12p70 levels was observed. Thus, neither *in vitro* induction nor *in vivo* levels of IL-12p70 and IL-18 appeared to cause the increased cytotoxic phenotype of intervillous MAIT cells. However, the levels of the anti-inflammatory cytokine IL-10 were higher in PB cultures compared to IVB and DP after *E*. *coli* stimulation. It can be speculated that the elevated levels of IL-10 could contribute to the lower MAIT cell activation in PB.

Previous studies have shown a limited cytolytic capacity of T cells at the foetal-maternal interface, a mechanism suggested to control robust pro-inflammatory immune responses to prevent preterm labour^44^. However, our data indicate that upon *E*. *coli* stimulation, MAIT cells from both IVB and DP were capable of expressing cytotoxic markers to similar or even greater levels than PB MAIT cells. Differences in proportion and function of antigen-presenting cells in blood and tissue could influence the response of MAIT cells. Decidual tissues contained a lower proportion of B cells and CD14^+^ myeloid cells compared to PB (Table 1), and yet decidual MAIT cells responded similarly. It has been shown that decidual tissues contain higher proportions of dendritic cells than PB^45^, and these could be potent antigen-presenting cells for MAIT cells. On the other hand, both macrophages and dendritic cells in decidua have been reported to have a tolerogenic phenotype^45, 46^, which may control T cell activation. The cytotoxicity of decidual T cells may also be reduced by trophoblast interactions^47^. Thus, *in vivo* the microenvironment may influence immune responses in the placental tissues.

The increased proportion of MAIT cells in IVB was accompanied by an increased number of B cells compared to PB, which have been shown to function as antigen-presenting cells for MAIT cells^49^. The phenotype of the B cells would need to be better defined to draw any conclusions about their role in the intervillous space, but it possible that they function as antigen-presenting cells to MAIT cells.

Syncytiotrophoblasts line the placental villi and are in direct contact with maternal IVB. They do not express polymorphic HLA molecules, and are devoid of expression of HLA-G. Our data indicate that they also do not express the MR1 molecule, diminishing their role as antigen-presenting cells to MAIT cells. The lack of MR1 expression in syncytiotrophoblasts may be an important mechanism for preventing excessive inflammatory responses by MAIT cells at the placental barrier. On the other hand, cells in the villous stroma expressed MR1 both at term and during early second trimester, which may be essential should there be an intra-uterine infection. The MR1^+^ cells in the villi were also expressing CD68, indicating that they were macrophages. It has been shown that maternal T cells, of which the majority were CD8^+^ T cells, can be detected inside the chorionic villi in villitis of unknown etiology^48^, a condition which affects over 10% of all pregnancies^49^. Acute and chronic villitis can also be caused by infections, of which viral infections are the most common^50^, but bacteria have also been reported to cause villous inflammation^51^. Thus, intervillous maternal MAIT cells could potentially interact with MR1-expressing placental macrophages, but also with B cells and monocytes on-site in the intervillous space during inflammatory conditions. MR1^+^ macrophages were also detected in DP, which may serve as antigen-presenting cells to decidual MAIT cells. However, the majority of the MR1^+^ cells were negative for CD68. Endometrial MR1^+^ cells have been shown to co-express HLA-DR or CD11c^37^, implying that they are dendritic cells and/or macrophages. It can therefore be speculated that the MR1^+^CD68^−^ cells in the decidua mainly consist of dendritic cells, and it is possible that some might be B cells even though they are scarce in decidual tissues.

Overall, these data indicate that some cellular components may reside in the intervillous space of the placenta, which hence does not only function as a site for exchange of oxygen, nutrients, and antibodies. Our findings suggest that MAIT cells at the foetal-maternal interface have the potential to exert a role in immune defence upon sensing specific antigens, as well as when exposed to proinflammatory cytokines. Further functional studies of MAIT cells and their interaction with trophoblasts and decidual stromal cells would be of interest to understand whether the microenvironment in the placental tissue affect their function. It also remains to be determined if MAIT cells are involved in preterm labour or other pregnancy complications.

## Material and Methods

### Sample collection

Human term placentas (median gestation week 39, range 38–42) were collected from healthy individuals (n = 34) (median age 34, range 21–42) giving birth through planned caesarean sections following healthy pregnancies. One additional placenta was obtained after elective termination at gestational week 13, which was induced by misoprostol. Written informed consent was obtained from the patients. The regional ethical committee of Karolinska Institutet approved the donation of placentas and peripheral blood samples (entry nos 2009/418-31/4, 2010/2061-32 and 2015/1848-31/2). All experiments were performed in accordance with relevant guidelines and regulations.

### Cell isolation

Paired samples of lymphocytes were isolated from PB, IVB, CB, and DP. PB was collected in heparin tubes a few hours prior to caesarean section. Damaged placentas presenting cuts or rifts were discarded. The umbilical cord was clamped at the end. To collect IVB, the placenta was washed in phosphate-buffered saline (PBS) and then lifted so that fresh blood from the intervillous space could drip down onto a petri dish from where it was quickly transferred to heparin tubes.

The DP was dissected from the amnion and chorion and cut into smaller pieces. Amnion tissue was washed and frozen at −80 °C for PCR analysis. The decidual tissue was washed in PBS by centrifugation at 600 g for 1 minute, and the supernatant was discarded. This step was repeated until the supernatant was clear. Decidual lymphocytes were released by mechanical disaggregation using a GentleMACS Dissociator (Miltenyi Biotec, Bergisch Gladbach, Germany). Following disaggregation, the cell suspension was consecutively filtered through a 100 μm metal mesh, and then through 70 μm and 40 μm cell strainers (VWR, Radnor, PA). After washing, the cells were re-suspended in RPMI supplemented with 0.1 mM ethylenediamine tetraacetic acid (EDTA, Sigma-Aldrich), layered on Lymphoprep (Axis-Shield, Dundee, Scotland) and centrifuged at 400 g for 30 min. The mononuclear cell layer was collected and washed three times in PBS.

The heparin tubes containing PB, IVB and CB were centrifuged at 600 g for 8 min and plasma samples were collected and frozen at −80 °C. Mononuclear cells were isolated by Lymphoprep gradient centrifugation.

### Cell cultures

All cultures were set up using freshly isolated cells. Mononuclear cells from PB, IVB and DP were incubated in 96-well plates at a concentration of 7.5 × 10^5^ cells in 250 μL/well in RPMI (HyClone, GE Health Sciences, South Logan, UT) medium supplemented with 10% foetal calf serum, 100 U/ml penicillin and 100 μg/ml streptomycin. The *E*. *coli* strain used in this study was a resident strain isolated from faecal samples from a child. It belonged to phylogenetic group B2 and expressed the following virulence factors: haemolysin, S-fimbriae type 1, and P fimbriae of papG class III^52^. The *E*. *coli* strain was fixed using 1% paraformaldehyde. Following titrations, fixed *E*. *coli* was added to the cultures using a multiplicity of infection (MOI) of 40 together with 1.25 μg/ml anti-CD28mAb (CD28.2, Biolegend, San Diego, CA) or left unstimulated. After 12 hours of culture at 37 °C and 5% CO_2_, 10 μg/ml Brefeldin-A (Sigma-Aldrich, St. Louis, MO) was added, followed by an additional 4 hours of culture. Supernatants were then collected and frozen at −80 °C before cells were harvested, washed and stained for flow cytometric analysis. For blocking experiments, 10 μg/ml anti-MR1 (26.5, Biolegend), 5 μg/ml anti-IL12p40 (C8.6, Biolegend), 5 μg/ml anti-IL18 (125–2 H, MBL, Woburn, MA) or the corresponding isotype controls were added to the cultures 1 hour before stimulation.

### Flow cytometry

Extracellular staining was performed in CliniMACS PBS/EDTA buffer (Miltenyi Biotech) supplemented with 0.1% bovine serum albumin. Intracellular staining was performed following extracellular staining using the BD Cytofix/Cytoperm^TM^ kit (BD Biosciences, Franklin Lakes, NJ) according to the manufacturer’s instructions. When only analysing extracellular markers, 7AAD was used to distinguish between live and dead cells. A list of the antibodies used in this study is shown in Supplemental Table 1. Data was collected using a BD FACSCanto flow cytometer and analysed with FlowJo software (Tree Star, Ashland, OR). Sub-gating was only performed when the parent gate contained more than 80 cells.

### ELISA

For IFN-γ, GrzB, and IL-12p70, ELISA kits (Mabtech, Stockholm, Sweden) were used according to the manufacturer’s instructions. For plasma sample analysis, ELISA diluent (Mabtech) was used for sample dilution. IL-18 was measured with IL-18 Duoset (R&D, Minneapolis, MN) according to manufacturer’s instructions. For IL-10, an in-house ELISA was set up, using an anti-human IL-10 coating antibody (127107, R&D), a secondary biotinylated goat anti-human IL-10 antibody (R&D), a standard of recombinant human IL-10 (R&D), and horseradish peroxidase (Sanquin, Amsterdam, Netherlands) for the enzymatic reaction.

### Fluorescence microscopy

Pieces of placental tissue (approximately 1 cm^3^) were snap frozen in liquid nitrogen and stored in −80 °C. The tissue was cut in a cryostat into 6 or 10 µm sections, mounted on glass and stored in −80 °C. The slides were fixed and permeabilised using a mixture of methanol (70%) and acetone (30%). Blocking was performed using Background Buster (Innovex biosciences, Richmond, CA). The sections were incubated with mouse anti-human MR1 (26.5, IgG2a,κ, Biolegend) and secondary Cy3-conjugated goat anti-mouse antibody (IgG, Jackson Immunoresearch, West Grove, PA) followed by biotinylated mouse anti-human CD68 (Y1/82 A, IgG2b,κ, eBioscience, Thermo Scientific, Waltham, MA), and DyLight488-conjugated streptavidin complex (Thermo Scientific). For the isotype control experiments in Supplementary Fig. S3, sections were stained with the mouse anti-human MR1 IgG2a,κ antibody or with IgG2a,κ isotype control (eBioscience), and with secondary Cy3-conjugated goat anti-mouse antibody, or with mouse anti-human CD68 (Y1/82 A, IgG2b,κ, Biolegend) or IgG2b,κ isotype control (eBioscience) followed an Alexa Fluor488-conjugated goat anti-mouse antibody (IgG, Thermo Scientific). DAPI (Nordic Biosite, Täby, Sweden) was used to visualize cell nuclei, and the sections were mounted in glycerol (Merck, Kenilworth, NJ).

An Olympus BX-51 fluorescence microscope was used, and pictures were taken with an Olympus XC30 camera. The data was collected and edited using CellSens standard software (Olympus, Tokyo, Japan). The camera settings from each sample was exported and used for the corresponding negative control.

### Statistical analysis

The non-parametric Friedman test was used to detect differences across three or more groups with repeated matched samples. If significant, the non-parametric Wilcoxon matched-pairs signed rank test was used for further testing between one group and another. For the post-tests, a Bonferroni corrected *P* value was used. To detect differences across two groups of matched samples, Wilcoxon matched-pairs signed rank test was used directly. For univariate tests, a two-tailed *P* value was used. Tests were performed using GraphPad Prism (GraphPad Software, La Jolla, CA). An alpha value of <0.05 was considered significant.

## Electronic supplementary material

Supplementary material
