# Supplementary material for: MAIT cells accumulate in placental intervillous space and display a highly cytotoxic phenotype upon bacterial stimulation
Source: Sci Rep. 2017 Jul 21;7:6123. doi: 10.1038/s41598-017-06430-6 (PMC5522401; doi:10.1038/s41598-017-06430-6)
Supplement: Supplementary file 1 — Supplementary material [file 41598_2017_6430_MOESM1_ESM.pdf]

# MAIT cells accumulate in placental intervillous space and display a highly cytotoxic phenotype upon bacterial stimulation

Martin Solders, Laia Gorchs, Tom Erkers, Anna-Carin Lundell, Silvia Nava, Sebastian Gidlöf, Eleonor Tiblad, Isabelle Magalhaes, and Helen Kaipe

## Supplementary material and methods

### Chimerism analysis

To evaluate the purity of the lymphocytes isolated from intervillous blood (IVB), samples from three donors were examined by microsatellite polymorphism. DNA was extracted from lymphocytes or from amniotic tissue using the EZ1 DNA blood kit and an EZ1 advanced XL instrument, or QIAmp DNA tissue kit (Qiagen, Hilden, Germany), respectively. DNA samples were amplified with four different short tandem repeats (STR) and analyzed using capillary electrophoresis (ABI 3130XL Genetic Analyzer; Applied Biosystems, Foster City, CA). If more than one STR was informative, the median of the results was used. Peripheral blood (PB) from the mother was used as a marker for maternal DNA, and amnion epithelia and cord blood (CB) were used as controls for fetal DNA. The amount of fetal DNA in the IVB samples was 5.2%, 11.0% and 13.2% respectively (representative plots in Supplementary Fig S1a).

To investigate the origin of MAIT cells in IVB, the cells were enriched using the Pan T cell Isolation Kit (Miltenyi Biotec, Bergisch Gladbach, Germany) according to the manufacturer's instructions. This was followed by staining with Phycoerythrin (PE)-conjugated anti-CD161 and subsequent positive selection using the Anti-PE Microbeads kit (Miltenyi Biotec) according to the manufacturer's instructions. DNA was then extracted and analyzed as

described above, using PB and CB as markers for maternal and fetal DNA, respectively. The amount of fetal DNA in the IVB samples was 8.6%, 14.0% and 19.4% before sorting (representative plots in Supplementary Fig S2a). After sorting, the amount of fetal DNA decreased to 5.8%, 9.5% and 10.5%, respectively.

Supplementary Figure S1

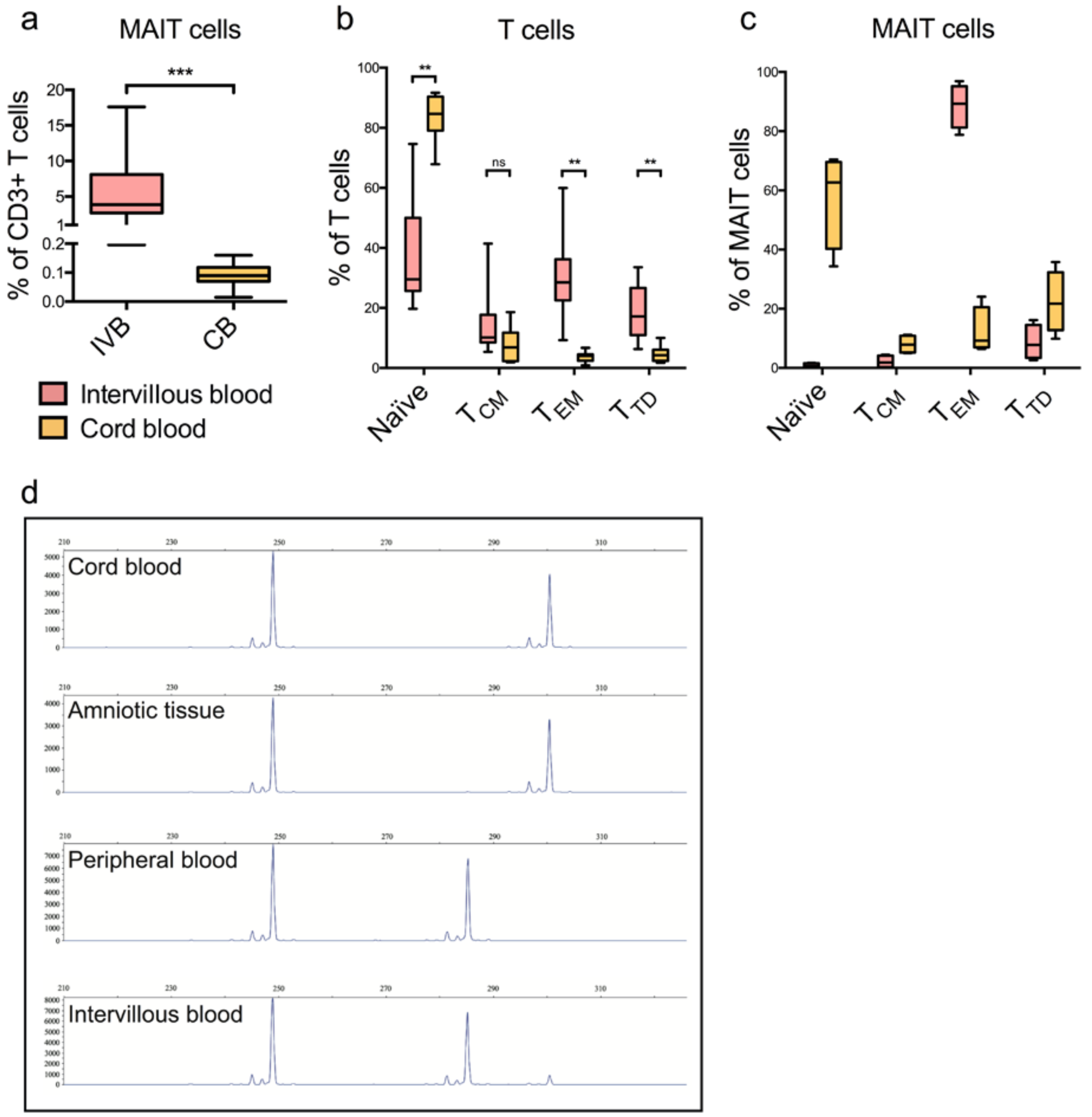


**Supplementary Figure S1. Low proportion of fetal MAIT cells in intervillous blood.** (**a-c**) Comparison of paired samples of intervillous blood IVB (light red) and umbilical cord blood (CB, yellow) samples. (**a**) The number of MAIT cells expressed as percentage of $CD3^+$ cells was lower in CB than in IVB (n = 19). (**b**) The T cell memory phenotype differed markedly between CB and IVB, with a large amount of naïve T cells in CB (n = 10). (**c**) The MAIT cell memory phenotype contrasted sharply between CB and IVB (n = 4). (**d**) One representative analysis out of three, measuring the amount of fetal DNA in intervillous blood (IVB). The Wilcoxon signed rank test was used to detect statistically significant differences across two groups of paired samples. ns = not significant, ***P* < 0.01, ****P* < 0.001.

## Supplementary Figure S2

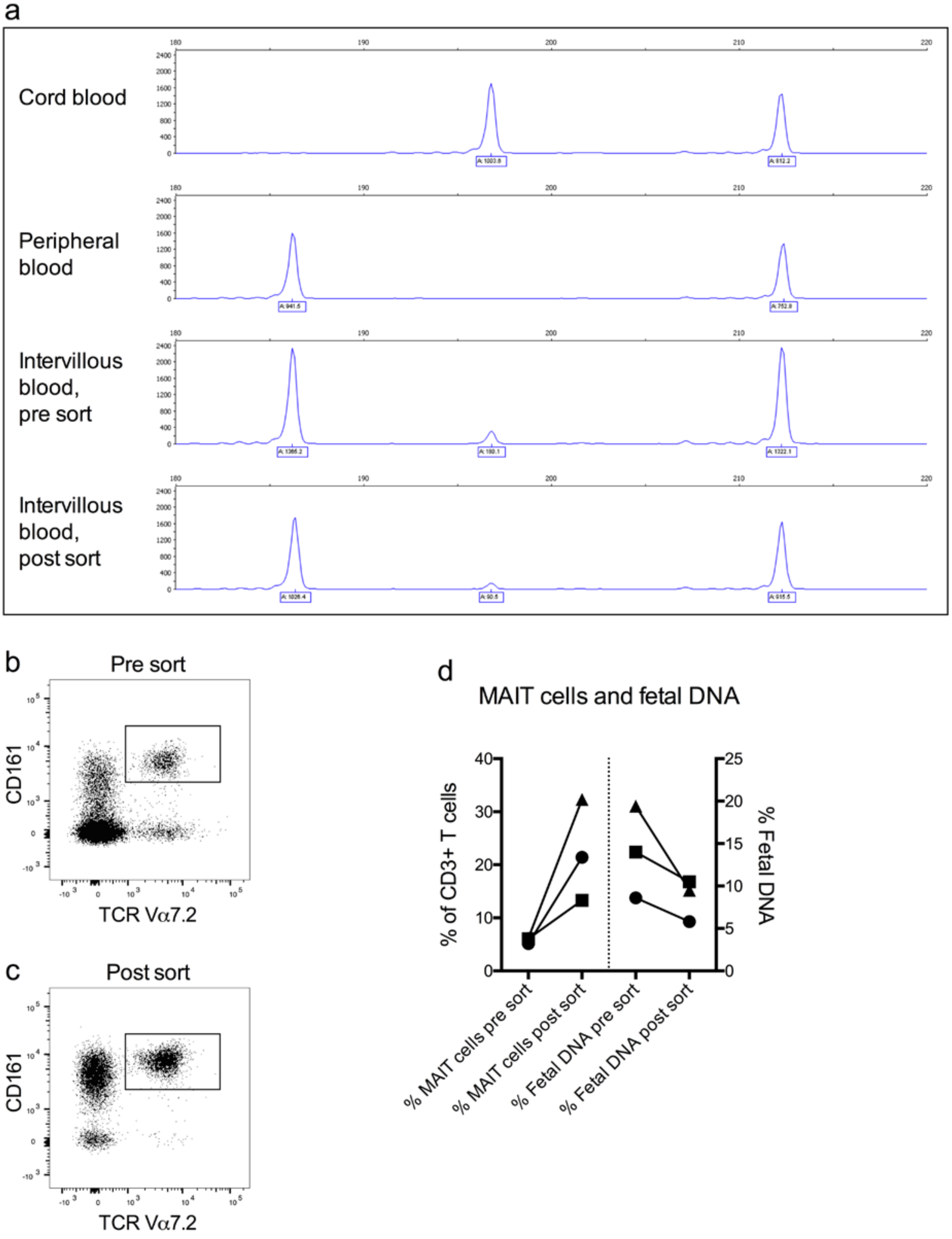

**Supplementary Figure S2. Enrichment of intervillous CD161$^+$ T cells decreased the amount of fetal DNA** (**a**) One representative analysis out of three, measuring the amount of fetal DNA in intervillous blood (IVB) before and after enrichment of CD161$^+$ T cells. Numbers below the peaks depict the calculated values for the area under curve. (**b-c**) Representative flow cytometry plots analyzing CD161 and TCR V$\alpha$7.2 expression on CD3$^+$ cells (CD4$^+$CD8$^+$ excluded) (**b**) before and (**c**) after enrichment. (**d**) The proportion of MAIT cells expressed as percentage of CD3$^+$ cells was increased in IVB following separation of CD161$^+$ T cells, whereas the amount of fetal DNA decreased (n = 3).

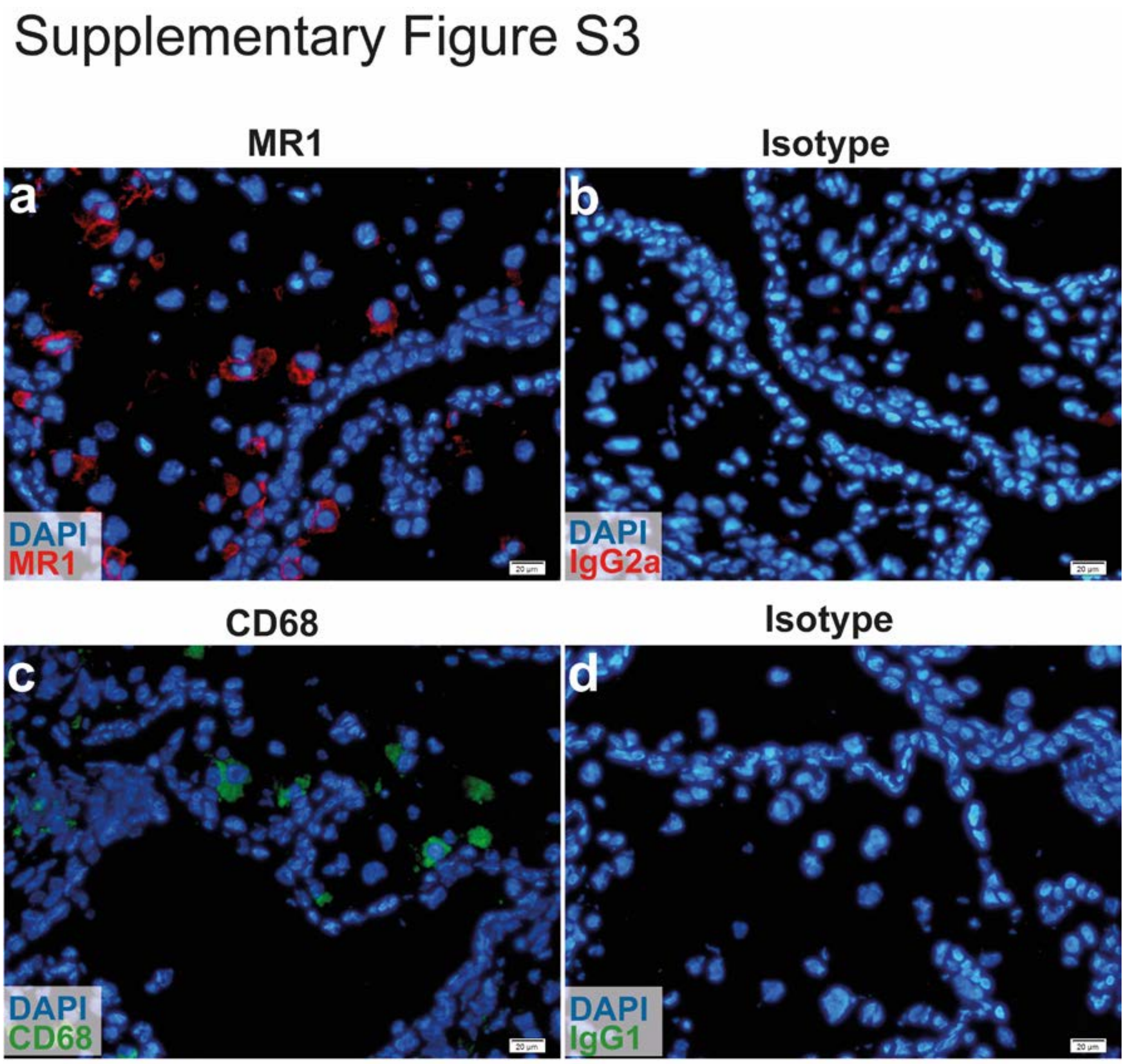


**Supplementary Figure S3. Immunofluorescence microscopic images with matching isotype controls.** Immunofluorescence microscopic images of placental tissue from gestation week 13 showing cross sections of chorionic villi and intervillous space stained with **(a)** MR1 (red) or (**c**) CD68 (green) or (**b**) and (**d**) the corresponding isotype controls at magnification × 20. All slides were stained with secondary goat anti-mouse antibodies, either Cy3- (**a-b**) or Alexa Fluor488-labelled (**c-d**). Nuclei were stained with DAPI (blue). The same microscopy settings were kept for the samples and the corresponding controls. Images showed specific staining for both MR1 and CD68 compared to the isotype control (*n* = 1).

Supplementary Table S1. **Antibodies and viability dye used for flow cytometry.**

| **Surface markers** | **Fluorochrome** | **Clone** | **Source** |
|---|---|---|---|
| CD3 | V450 | UCHT1 | BD |
| CD3 | FITC | UCHT1 | BD |
| CD3 | PE-Cy7 | UCHT1 | BD |
| CD4 | V500 | RPA-T4 | BD |
| CD8 | A700 | RPA-T8 | BD |
| CD8 | APC | SK1 | BD |
| CD8 | PE-Cy7 | RPA-T8 | BD |
| CD14 | FITC | MϕP9 | BD |
| CD16 | PE | 3G8 | BD |
| CD19 | PE-Cy7 | SJ25C1 | BD |
| CD20 | A700 | 2H7 | BD |
| CD25 | Bv421 | M-A251 | BD |
| CD38 | Bv421 | HIT2 | BD |
| CD45 | APC-H7 | 2D1 | BD |
| CD45RA | PE-Cy7 | HI100 | BD |
| CD56 | APC | NCAM16.2 | BD |
| CD69 | FITC | L78 | BD |
| CD127 | APC-A700 | R34.34 | Beckman Coulter |
| CD161 | PE | HP-3G10 | BioLegend |
| CD197 (CCR7) | PE-CF594 | 150503 | BD |
| CD279 (PD-1) | BV421 | EH12.1 | BD |
| HLA-DR | FITC | G46-6 | BD |
| TCR Vα7.2 | APC-Cy7 | 3C10 | BioLegend |
| - | 7AAD | - | BD |
| **Intracellular markers** | | | |
| Granzyme B | FITC | GB11 | BD |
| IFN-γ | PE-Cy7 | 4S.B3 | BD |
| IFN-γ | PE-Cy7 | B27 | BD |
| IL-17 | A488 | N49-653 | BD |
| IL-22 | APC | REA466 | Miltenyi |
| Ki-67 | A488 | B56 | BD |
| Perforin | A647 | δG9 | BD |

Abbreviations; CD, Cluster of differentiation, BD, BD Biosciences (Franklin Lakes, NJ), FITC, Fluoresceinisothiocyanate, Pe-Cy7, Phycoerythrin-cyanine 7, A700, Alexa Fluor 700, APC, Allophycocyanine, PE, Phycoerythrin, BV421, Brilliant violet 421, APC-H7, Allophycocyanine H7, APC-A700, Allophycocyanine Alexa Fluor 700, Beckman Coulter, Beckman Coulter (Fullerton, CA), BioLegend, Biolegend (San Diego, CA), PE-CF594, Phycoerythrin-CF 594, APC-Cy7, Allophycocyanine-indo tricarbocyanine, Miltenyi, Miltenyi Biotec (Bergisch Gladbach, Germany), A488, Alexa Fluor 488, A647, Alexa Fluor 647.
